# Optimisation of Active Magnetic Elements in Beam-like Structures—Numerical Modelling Studies

**DOI:** 10.3390/ma17194929

**Published:** 2024-10-09

**Authors:** Katarzyna Majewska

**Affiliations:** Institute of Fluid Flow Machinery, Polish Academy of Sciences, Fiszera 14, 80-231 Gdansk, Poland; k.majewska@imp.gda.pl

**Keywords:** optimisation, magnetic shape memory actuator (MSM actuator), magnetorheological damper (MR damper), classical shape memory wire (SM wire), beam-like structure

## Abstract

This paper explores integrating advanced materials, including magnetic shape memory alloys, magnetorheological fluids, and classical shape memory alloys, within structural elements to achieve exceptional physical properties. When these materials are integrated within structures—whether as wires, actuators, or dampers—they provide the structures with unique static, dynamic, and damping characteristics not commonly found in nature. This study aimed to evaluate the efficacy of these active materials in enhancing the performance of beam-like structures. This investigation was conducted through a comprehensive numerical analysis, focusing on a composite beam. The study examined the impact of different active elements, their position within the structure, and their influence on key dynamic properties. Additionally, a simplified damage scenario was considered, wherein the adverse effects of structural damage were mitigated through the strategic application of these materials. Numerical simulations were carried out using the finite element method, with custom computational codes developed in MATLAB. The findings of these simulations are presented and discussed in this paper.

## 1. Introduction

Today, various smart, intelligent, adaptive, and multifunctional materials are drawing significant attention from the international scientific community. These materials possess one or more properties that can be significantly altered by external stimuli, such as stress, temperature, moisture, pH levels, and electric or magnetic fields. Smart materials can be categorized into several main groups, each defined by the specific mechanisms through which they respond to these stimuli [1,2,3,4]:

- Colour-changing materials—alter colour in response to changes in environmental conditions such as temperature, pH, and light. Applications: temperature indicators, dynamic displays, camouflage, and environmental monitoring.
- Light-emitting materials—emit light when excited by an external energy source, such as electrical current and ultraviolet light. Applications: displays, signage, lighting, and decorative elements.
- Moving materials—can change shape or move in response to external stimuli such as heat, light, and electrical fields. Applications: actuators, robotics, and adaptive structures.
- Temperature-changing materials—change thermal properties, such as thermal conductivity and heat capacity, in response to temperature changes. Applications: building insulation, thermal management in electronics, and temperature-controlled packaging.
- Thickness-changing fluids—change viscosity or thickness in response to external stimuli such as magnetic and electric fields. Applications: adaptive suspension systems, vibration control, and precision manufacturing.
- Self-assembling materials—spontaneously form structured patterns or structures at the microscopic or macroscopic level without external guidance. Applications: nanotechnology, drug delivery systems, and advanced manufacturing.
- Self-repairing materials/self-healing materials—can autonomously repair themselves after damage, restoring their original properties. Applications: structural materials, coatings, and infrastructure, including self-healing roads and buildings.

These materials represent a broad range of capabilities and applications, showcasing the versatility and innovation within the field of smart materials.

The unique properties of these materials have led to numerous applications, and their controllable characteristics make them highly valuable in scientific research. Consequently, materials such as piezoelectric materials, electro- and magnetorheological fluids, and thermally and magnetically activated shape memory alloys have remained a focal point of study for many years and continue to attract significant interest.

**Figure 1:**
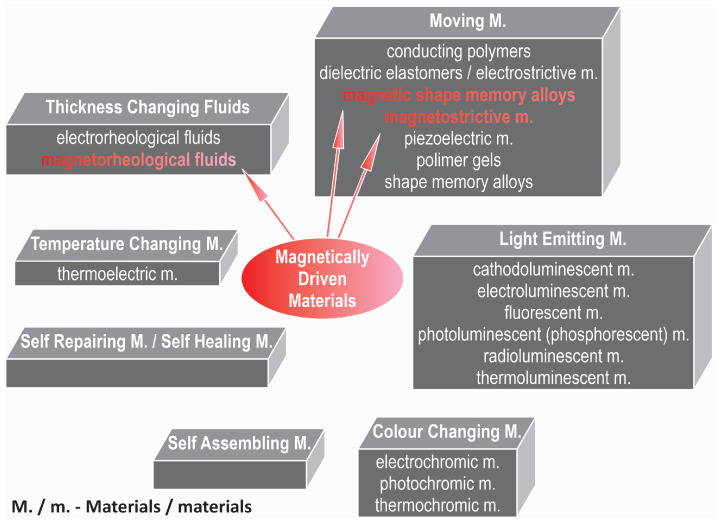
Main groups of smart materials based on [1,2].

In Figure 1, the aforementioned groups of materials are presented, with a particular focus on those of interest to the author—specifically, magnetically driven materials, such as magnetic shape memory alloys (MSMA) and magnetorheological (MR) fluids.

The paper investigates the application of smart materials in control processes, demonstrated through a case study involving a layered cantilever composite beam. Initially, the paper presents the material selection process, followed by a review of preliminary numerical research [5]. The research involves a comprehensive analysis of various parameters, using finite element method (FEM) simulations, to assess the impact of various intelligent material actuators on beam vibrations. This includes testing different configurations of shape memory (SM) alloy wires, magnetic shape memory (MSM) actuators, and magnetorheological (MR) fluids. Key findings show that SM wires significantly reduce beam vibrations when the outer layers are fully composed of SM alloy wires. MSM actuators offer the greatest vibration reduction with specific excitation amplitudes and frequencies, while MR dampers are most effective when fully activated. Overall, the study demonstrates how these smart materials can enhance dynamic control in composite beam structures. The next section of the paper focuses on magnetically driven devices (MSM actuator and MR damper), with particular emphasis on identifying the optimal placement of these active elements for both undamaged and damaged beam-like structures. Concluding remarks are then presented.

## 2. Material Selection—Why Magnetically Driven?

Many papers in the available literature focus on smart materials’ development, properties, and applications. To highlight the research gap, magnetic shape memory alloys and magnetorheological materials were compared with piezoelectric (PZT) materials using the following keywords: effect, material, device, and rotor.

**Figure 2:**
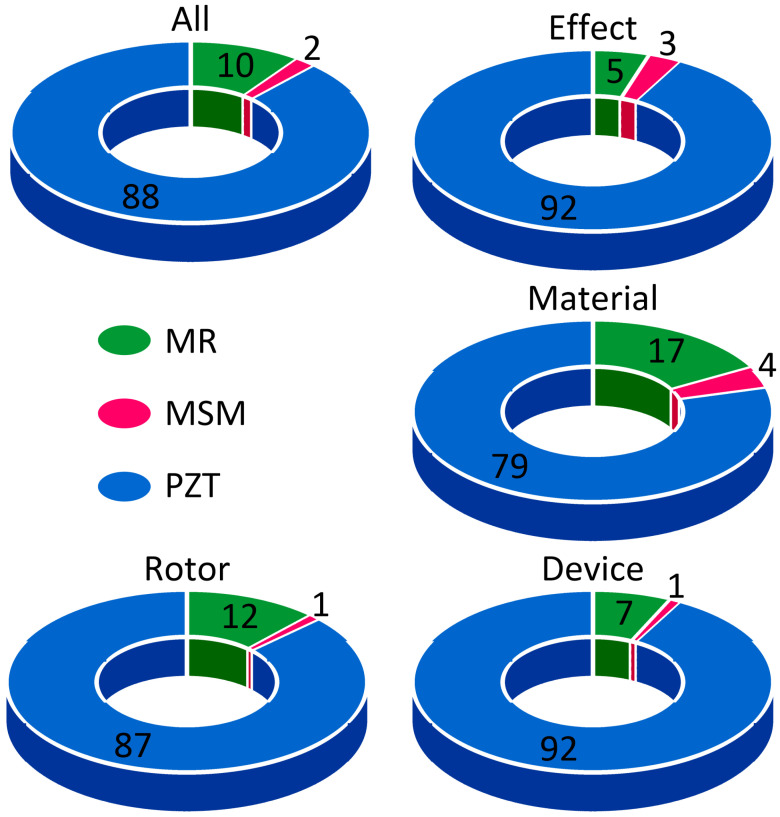
Comparison of the number of papers (in %), related to the researcher’s interest, published in the last decades.

As shown in Figure 2, PZT has the highest number of publications and citations, reflecting its widespread use and extensive research history. Although MSM and MR materials have fewer publications, they rapidly expand as their applications become more diverse and innovative. PZT research often focuses on practical applications and device performance, while MSM and MR materials are investigated for their unique material properties and potential in advanced adaptive systems. The research emphasis for MSM and MR materials/devices increasingly centres on optimizing their properties for specific applications, such as robotics, adaptive structures, and advanced damping systems, reflecting a trend towards integrating these materials into cutting-edge technologies. Overall, the statistical trends indicate that while PZT dominates research volume and application breadth, MSM and MR materials/devices are emerging fields with growing interest, particularly in specialized and innovative applications. Additionally, there remains a notable gap in the available literature regarding the optimisation of the placement of magnetic (MSM, MR) materials in a structure/construction.

### 2.1. Magnetic Materials—Short Description

Magnetic shape memory alloys, also known as ferromagnetic shape memory (FSM) alloys, are materials that undergo significant, completely reversible, nonlinear deformation when driven by an external magnetic field. In MSM alloys, the application of a magnetic field causes a rearrangement of the internal crystal structure, known as martensitic variants, leading to a macroscopic shape change. The magnetic shape memory effect (MSME) in MSM alloys refers to the large nonlinear strain induced in the martensitic phase of the alloys upon exposure to an external magnetic field. This effect was first observed by Ullakko in 1996 [6]. The effect can be observed in several alloys, like Iron–Palladium (FePd), Cobalt–Nickel–Gallium (Co-Ni-Ga), and Nickel–Iron–Gallium (NiFeGa). However, the most relevant MSM alloy is the Nickel–Manganese–Gallium (NiMnGa) alloy together with its derivates, which exhibits giant magnetostrains of up to 12% [7,8,9]. Because magnetic fields can be applied and removed rapidly, devices made out of MSM alloys are suitable for high-speed actuation, dynamic applications, and various advanced technologies. They are particularly useful in scenarios requiring fast, precise movement and control, such as robotics [10], vibration control [11,12], sensors [13], actuators [14], and medical devices [15].

Magnetorheological materials, particularly MR fluids, are smart materials that exhibit significant changes in their rheological properties in response to an external magnetic field. These materials consist of magnetic particles, typically iron-based, suspended in a non-magnetic carrier fluid. When exposed to a magnetic field, the particles align along the field lines, causing an increase in the fluid’s viscosity and shear modulus. This phenomenon, known as the magnetorheological effect, enables the material to transition from a liquid-like to a nearly solid-like state. MR fluids were first developed by Jacob Rabinow in 1948 [16,17]. One of their key characteristics is their ability to undergo rapid and reversible changes in mechanical properties. The response time to a magnetic field is typically on the order of milliseconds, making these materials ideal for applications that require quick performance adjustments. When the magnetic field is removed or altered, the material swiftly returns to its original state. Due to their versatility, MR fluids are used in a wide range of applications. These include automotive systems for adaptive suspensions [18], shock absorbers and vibration control [19], as well as prosthetics, where they are employed to provide adjustable stiffness [20].

### 2.2. Other Materials—Short Description

Thermally (classically) activated shape memory alloys are a class of smart materials capable of undergoing significant and reversible deformation in response to changes in temperature. These materials are typically composed of Nickel–Titanium (NiTi) alloys. The defining feature of SM alloys is their ability to *remember* a pre-defined shape, which they return to when exposed to a specific thermal stimulus. This behaviour is driven by a solid-state phase transformation between two crystal structures: martensite (the low-temperature phase) and austenite (the high-temperature phase). The alloy remains in the martensitic phase at low temperatures, where it can be easily deformed. The alloy transforms into the austenitic phase upon heating, causing it to revert to its original shape. This process is fully reversible, allowing for the material to return to the martensitic phase and be reshaped once it cools down [21]. The first discovery of the shape memory effect (SME) occurred in 1932 [22], with NiTi (Nitinol) being patented in 1962 [23]. SM alloys are renowned for their unique thermomechanical properties, including high strain recovery, durability, and biocompatibility. These characteristics make them ideal for a wide range of applications, including medical devices, such as stents and *guidewires* [24], actuators in aerospace and robotics [25], and temperature-sensitive mechanisms like thermal valves and safety devices [26].

All of the above-mentioned materials/devices were integrated into beam-like structures made out of composite materials. Composite materials contain two or more distinct constituent phases, usually involving a matrix (such as polymer, metal, or ceramic) and a reinforcement (such as fibres, particles, and layers). These phases retain their individual properties but work together to enhance the overall performance of the material [27]. Therefore, one of the primary advantages of composite materials is their ability to be tailored, offering remarkable design flexibility. This feature allows for composites to be formed into complex shapes that are sometimes impossible to achieve using traditional materials. Composite materials tend to have good fatigue resistance, as they can withstand cyclic loading for extended periods without failure. Composite materials are widely used across multiple industries, such as aerospace [28], automotive [29], sports equipment [30], marine [31], civil engineering [32], and medicine [33].

## 3. Structural Dynamic Control by Selected Active Elements

This section aims to examine the potential of intelligent materials, particularly magnetic ones, for optimising their placement and control mechanisms. To illustrate it, a layered cantilever composite beam was selected for the study.

### Geometry Definition

**Figure 3:**
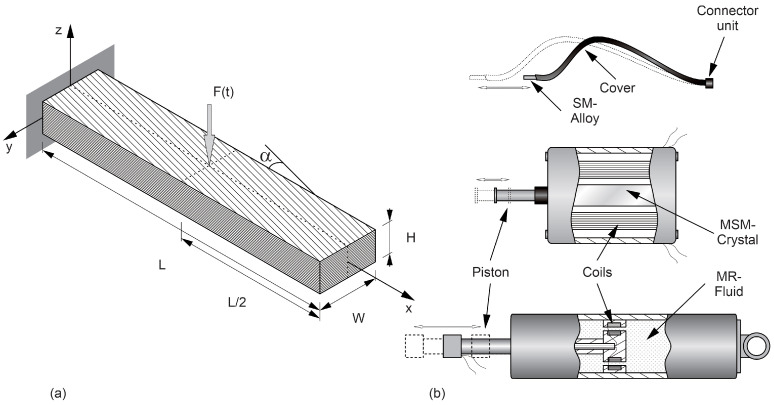
(**a**) Object of investigation with geometry, (**b**) structure and working principle of actuators.

The geometrical dimensions of the beam are presented in Figure 3a. The beam measures 500 mm in length (L), 20 mm in width (W), and 10 mm in height (H). Figure 3b illustrates the working principles of the SM wire, MSM actuator, and MR damper, as well as the basic construction of each.

The beam consists of 20 layers of a glass/epoxy composite material, with the orientation of the reinforcing glass fibres defined by a [(45)_10_] stacking sequence. The beam is clamped at one end, with SM alloy wires integrated into the structure for actuation. Alternatively, an MSM actuator or MR damper is attached at the other end (see Figure 3b for a detailed view of the wire, actuator, and damper). The SM wire, MSM actuator, and MR damper operate based on the unique property of a certain medium. This means that the SM wire deforms at lower temperatures, and the wire undergoes a martensitic phase transformation, allowing it to bend or be elongated. Upon heating, it returns to its original shape due to a reverse phase change to austenite, recovering its form and generating force. The MSM actuator undergoes a reversible change in shape in response to magnetic fields, allowing the actuator to convert magnetic energy directly into mechanical motion. The applied magnetic field induces a shift in the atomic structure, causing a reversible mechanical deformation that can be used to drive the actuator. Meanwhile, the MR damper works by utilizing a fluid with suspended magnetic particles. When exposed to a magnetic field, these particles align and change the fluid’s viscosity, allowing the damper to adjust its resistance to movement.

The average properties of the composite material components collected from available sources are provided in Table 1, and the parameters of the SM wire, MSM actuator, and MR damper are provided in Table 2.

## 4. Results and Discussion

### 4.1. Numerical Simulation

For the numerical analysis, the finite element method (FEM) was employed using a custom MATLAB program. A finite element model consisting of 50 elements was used to analyse the dynamic behaviour of a composite beam. Each element was represented by three nodes, with three degrees of freedom assigned to each node. As a result, the total system comprised 450 degrees of freedom. The Newmark method was applied for numerical integration to solve the equations of motion, ensuring stability and accuracy in identifying the beam’s natural frequencies and mode shapes. The model included clamped boundary conditions at one end, limiting transverse displacement and rotation. Wires, an actuator, and a damper were added as excitation sources (force applied at specific nodes in the finite element model of the beam) to investigate the effects of external forces. Material properties, such as Young’s moduli and densities of the composite layers, were also included for a thorough analysis of the beam’s dynamic characteristics.

In each simulation, an external harmonic force F(t) = sin(ωt) with an amplitude of F*_Q_* = 1 N and a frequency of f*_Q_* = 5 Hz was applied at the mid-span of the beam, while the beam’s responses were measured along its entire length. The beam was assumed to be clamped at one end. The results, including the first six natural frequencies (19.3 Hz, 120.5 Hz, 335.5 Hz, 652.0 Hz, 1066.4 Hz, and 1572.8 Hz) and their corresponding vibration modes, are detailed in Table 3.

### 4.2. Influence of SM Wires, MSM Actuators, and MR Dampers on Selected Dynamic Characteristics

This section presents a brief review of previous work [5]. It examines in detail the influence of SM wires, MSM actuators, and MR dampers on the dynamic characteristics of an undamaged, layered cantilever beam. The colours in the figures in Section 4.2 are defined as follows: red/blue—higher vibrations of the beam; yellow/green—higher reduction in vibrations of the beam.

#### 4.2.1. Beam with SM Alloy Wires

The investigation was conducted to study the behaviour of a beam with specified geometrical and mechanical properties (see Figure 3, Table 1). The only modification made to the beam’s structure was the addition of SM Nitinol wires (see Table 2) as reinforcement in the outer layers. Consequently, the stacking sequence of the beam was adjusted to [0, (±45)_9_, 0]. The beam was subjected to a harmonic excitation force with an amplitude of F*_Q_* = 1 N at the excitation frequency of f*_Q_* = 5 Hz. Changes in the beam’s vibrations were then observed.

**Figure 4:**
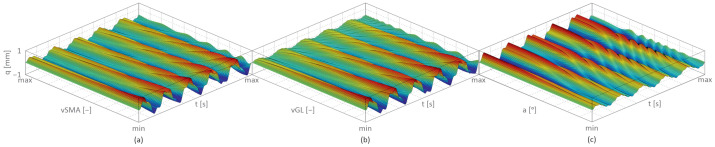
Transverse displacement as a function of time: (**a**) SM alloy wires’ volume fraction (min = 0%, max = 100%), (**b**) reinforcement glass fibres’ volume fraction (min = 0%, max = 100%), and (**c**) angle of reinforcement fibre (min = 0°, max = 90°) based on [5].

Figure 4 presents the transverse displacement (q) as a function of changes in the SM alloy wires’ volume fraction (vSMA)—Figure 4a; the reinforcement glass fibres’ volume fraction vGL—Figure 4b; and the angle a of the reinforcement glass fibres—Figure 4c.

The results presented in Figure 4 for the undamaged, layered cantilever beam with SM alloy wires in both outer layers show a significant vibration reduction under specific conditions. The greatest reduction occurred when the outer layers were entirely made out of SM alloy wires, with a relative volume fraction of 100% (max). Similarly, the inner layers, entirely made of glass fibres, also had a 100% (max) relative volume fraction. Also, the SM alloy wires were aligned parallel to the beam’s longitudinal axis at a 0° reinforcement angle (min).

#### 4.2.2. Beam with MSM Actuator

The investigation was conducted to study the behaviour of a beam with specified geometry and mechanical properties (see Figure 3, Table 1). However, it was assumed that the beam was clamped at one end, while its second end was supported by the MSM actuator (see Table 2). The beam was subjected to a harmonic excitation force with an amplitude of F*_Q_* = 1 N at the excitation frequency of f*_Q_* = 5 Hz. Simultaneously, the MSM actuator was excited with an amplitude of $F_{Q_{MSM}}$ = 0.5 N at the excitation frequency of $f_{Q_{MSM}}$ = 5 Hz. Changes in the beam’s vibrations were then observed.

**Figure 5:**
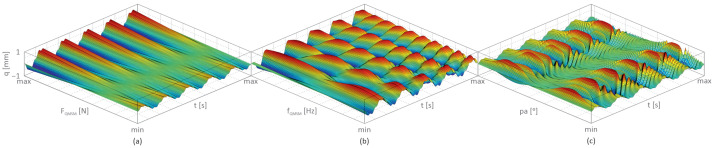
Transverse displacement as a function of time: (**a**) MSM actuator’s excitation amplitude (min = 0 N, max = 1 N), (**b**) MSM actuator’s excitation frequency (min = 0 Hz, max = 10 Hz), and (**c**) MSM actuator’s phase angle (min = 0°, max = 360°) based on [5].

Figure 5 presents the transverse displacement (q) as a function of changes in the MSM actuator’s excitation amplitude $F_{Q_{MSM}}$—Figure 5a; the MSM actuator’s excitation frequency $f_{Q_{MSM}}$—Figure 5b; and the MSM actuator’s phase angle pa—Figure 5c.

The results presented in Figure 5 for the undamaged, layered cantilever beam with an MSM actuator reveal that the highest reduction in beam vibrations was achieved when the excitation amplitude of the MSM actuator was equal to $F_{Q_{MSM}}$ = 0.4 N ($F_{Q_{MSM}}$ varies from 0 N to 1 N), when the MSM actuator’s excitation frequency $f_{Q_{MSM}}$ was up to 5 Hz ($f_{Q_{MSM}}$ varies from 0 Hz to 10 Hz), and when the MSM actuator’s phase angle pa was close to 180° (pa varies form 0° to 360°).

#### 4.2.3. Beam with MR Damper

The investigation was conducted to study the behaviour of a beam with specified geometrical and mechanical properties (see Figure 3, Table 1). However, it was assumed that the beam was clamped at one end, while its second end was supported by the MR damper (see Table 2). The beam was subjected to a harmonic excitation force with an amplitude of $F_{Q}$ = 1 N at an excitation frequency of $f_{Q}$ = 5 Hz. Simultaneously, the MR damper, as previously the MSM actuator, was excited with an amplitude of $F_{Q_{MSM}}$ = 0.5 N at an excitation frequency of $f_{Q_{MSM}}$ = 5 Hz. Changes in the beam’s vibrations were then observed.

**Figure 6:**
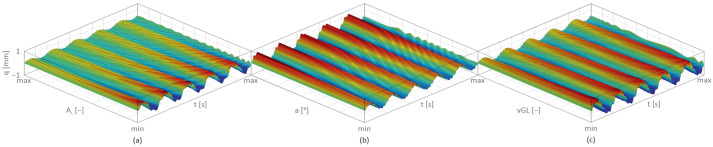
Transverse displacement as a function of time: (**a**) MR damper activation level (min = 0%, max = 100%), (**b**) angle of reinforcement glass fibre (min = 0°, max = 90°), and (**c**) reinforcement fibre volume fraction (min = 0%, max = 100%); based on [5].

Figure 6 presents the transverse displacement (q) as a function of changes in the MR damper activation level $A_{L}$ —Figure 6a; the angle a of reinforcement glass fibre—Figure 6b; and the reinforcement glass fibre volume fraction vGL—Figure 6c.

The results presented in Figure 6 for an undamaged, layered cantilever beam with the MR damper show that the greatest reduction in beam vibrations occurs when the MR damper is fully activated (i.e., 100% (max)). Also, this effect is most visible when the reinforcement angle is 0°, meaning the reinforcing glass fibres are parallel to the beam’s longitudinal axis, and when the relative volume fraction of the reinforcement is 100% (max), meaning the entire beam is composed of glass.

### 4.3. Optimisation of MSM Actuator and MR Damper Position

Optimising the placement and performance of MSM actuators and MR dampers in beam-like structures is essential for controlling vibrations and improving overall system stability and performance. This need for effective vibration control extends to various real-life technologies, such as automotive applications, where implementing strategic optimisation is crucial for enhancing suspension performance and improving vehicle ride comfort and stability [37]. In the context of buildings, damping systems significantly improve the safety of closely located structures during seismic events, further enhancing their overall performance [38]. Also, in the field of implants and biomaterial development, using magnetic fields during and after the insertion of devices like stents and clamps allows for precise adjustments to their fit and functionality, ensuring better integration with the body [39]. Therefore, MSM actuators should be positioned where the deformation is large, as their purpose is often to control or modify the beam’s response to external loads. MR dampers, designed to dissipate energy, should be placed at points where high stresses and dynamic loads are present. This optimisation process involves simulating different configurations using finite element analysis, validating the results experimentally, and fine-tuning settings based on real performance and adaptive control strategies. The proper placement of these devices ensures effective mitigation of unwanted deformation and vibrations, enhancing structural integrity, durability, and efficiency. This approach significantly improves the beam’s dynamic behaviour by reducing vibrations and dissipating energy. Here, the results of optimisation, using finite element analysis for the application of MSM actuators and MR dampers in beam-like structures, are presented. This paper not only focuses on optimising the placement of an MSM actuator and MR damper in both undamaged and damaged states of beams, but also explores other related aspects based on previous research.

For analysing the dynamic responses of a composite beam and visualizing the results, a key indicator, the Relative Vibration Energy ($E_{R}$), was proposed. This indicator serves as a specific measure of relative vibration energy to evaluate the efficiency of vibration control mechanisms (ratio of the energy associated with the vibrations of a structure relative to a baseline/reference state). To calculate $E_{R}$, the total vibration energy E($i_{1}$, $i_{2}$) for each configuration is first determined by summing the squared magnitudes of the Fourier coefficients obtained from the system’s response:

(1) $$E\left(i_{1},i_{2}\right)=\sum_{f}\sum_{k}{\left|Z\left(f,k\right)\right|}^{2}$$

where Z(f, k) denotes the complex amplitude at frequency f and spatial index k. This energy is then normalized against the maximum total energy observed across all tested configurations:

(2) $$s\left(i_{1},i_{2}\right)=\frac{E(i_{1},i_{2})}{max(E)}$$

where max(E) represents the maximum total vibration energy across all configurations. The $E_{R}$ is computed as

(3) $$E_{R}=1-s\left(i_{1},i_{2}\right)$$

The colours in the figures in Section 4.3.1 and Section 4.3.2 are defined as follows: red—maximum of $E_{R}$; blue—minimum of $E_{R}$.

#### 4.3.1. Undamaged State

As the first case, the investigation focused on an undamaged, clamped beam equipped with an MSM actuator. The beam was subjected to vibration by an external harmonic force F(t) = sin(ωt), with an amplitude $F_{Q}$ = 1 N and frequency of $f_{Q}$ = 5 Hz. The MSM actuator was operated at a frequency $f_{Q_{MSM}}$ = 5 Hz, with a phase shift of φ = 180° relative to the harmonic force F(t). The position of the MSM actuator was varied along the length of the beam, moving from one end to the other while simultaneously adjusting the excitation amplitude of the MSM actuator $F_{Q_{MSM}}$, from 0 N to 1 N.

**Figure 7:**
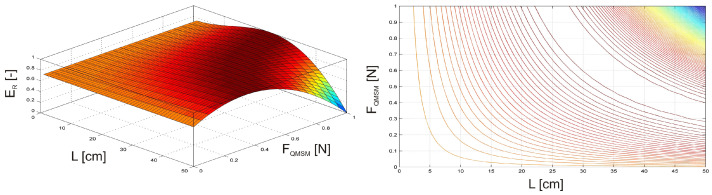
Relative vibration energy as a function of MSM actuator position and excitation amplitude.

It is visible in Figure 7 that the maximum relative vibration energy $E_{R}$ occurs when the MSM actuator is positioned between the excitation force F(t) (see Figure 3) and the free end of the beam, with the excitation amplitude $F_{Q_{MSM}}$ ranging from 0.35 N to 1 N.

As the second case, the investigation focused on an undamaged, clamped beam equipped with an MR damper. To ensure consistent excitation conditions, the beam was subjected to vibration by an external harmonic force F(t) = sin(ωt) with an amplitude $F_{Q}$ = 1 N and frequency of $f_{Q}$ = 5 Hz. The position of the MR damper was varied along the length of the beam, moving from one end to the other while simultaneously adjusting the MR damper’s activation level $A_{L}$ from 0 to 1, with 1 representing full activation of the MR damper.

**Figure 8:**
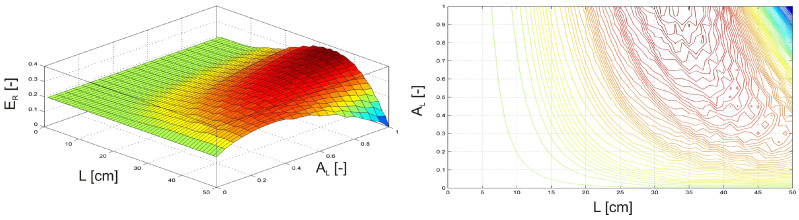
Relative vibration energy as a function of the MR damper position and its activation level.

In this example (Figure 8), the maximum relative vibration energy $E_{R}$ corresponds to the MR damper located between the excitation force F(t) and the free end of the beam and when its actuation level is 80–100%.

In the above cases, the investigation focused on an undamaged, clamped beam subjected to external harmonic vibrations. In both cases, the maximum relative vibration energy $E_{R}$ is observed when the magnetically driven device is placed between the excitation force and the free end of the beam.

#### 4.3.2. Damaged State

Following the above analysis, a simple damage scenario was explored to assess its impact while minimizing its negative effects. Damage was simulated by altering the beam’s material properties in the “plastic zone”, representing a local reduction in stiffness. This approach characterizes damage as a degradation in material stiffness that affects the beam’s dynamic response under load. Four specific examples were prepared to illustrate how an MSM actuator and an MR damper can mitigate the adverse effects of damage, demonstrating their potential to enhance the performance of beam-like structures.

**Figure 9:**
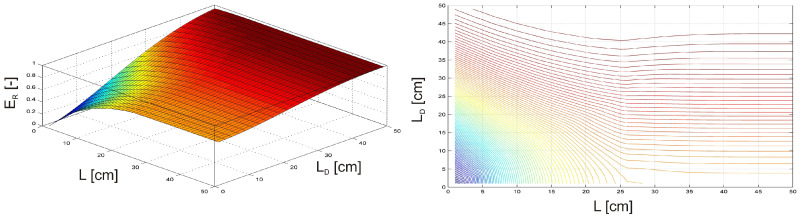
Relative vibration energy as a function of the MSM actuator and damage position.

**Figure 10:**
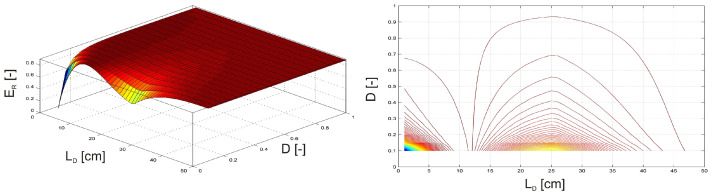
Relative vibration energy as a function of damage position and intensity of damage plasticity.

In the first two examples, the investigation focused on a damaged, clamped beam equipped with an MSM actuator. F(t) = sin(ωt) of the amplitude $F_{Q}$ = 1 N and frequency $f_{Q}$ = 5 Hz. The MSM actuator was excited with an amplitude $F_{Q_{MSM}}$ = 0.35 N, a frequency $f_{Q_{MSM}}$ = 5 Hz, a phase shift φ = 180° relative to the harmonic force F(t). The position of the MSM actuator and the damage position $L_{D}$ were varied along the length of the beam (Figure 9). Additionally, the intensity of the damage plasticity D was adjusted from 0 to 1 (Figure 10), where 1 indicates a fully broken beam.

In Figure 9, the maximum relative vibration energy $E_{R}$ is observed when the MSM actuator and damage are positioned between the excitation force F(t) and the free end of the beam, although not at the same location. Conversely, Figure 10 shows that the maximum relative vibration energy $E_{R}$ occurs when the damage is located either between the clamped end and the excitation force F(t) or between the excitation force F(t) and the free end of the beam, provided that the intensity of damage plasticity does not exceed 50%.

**Figure 11:**
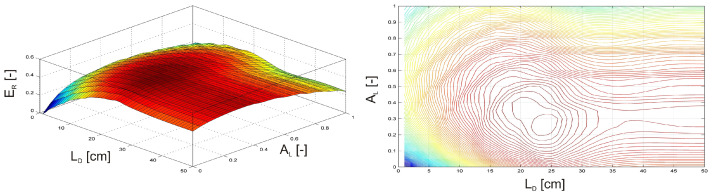
Relative vibration energy as a function of damage position and the MR damper activation level.

**Figure 12:**
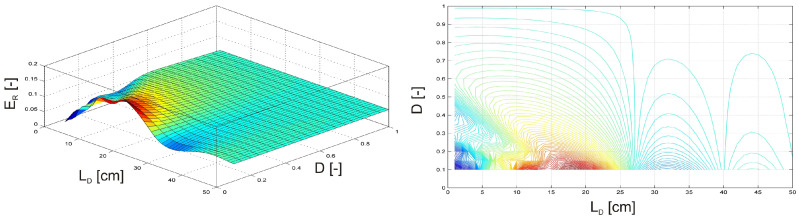
Relative vibration energy as a function of damage position and intensity of damage plasticity.

In the next two examples, the investigation focused on a damaged, clamped beam equipped with an MR damper subjected to an external harmonic force F(t) = sin(ωt) with an amplitude of F*_Q_* = 1 N and a frequency of f*_Q_* = 50 Hz. In the first scenario (Figure 11), the MR damper was attached to the free end of the beam, while the damage position L*_D_* was varied along the beam. Simultaneously, the activation level of the MR damper, A*_L_*, was adjusted from 0 (inactive) to 1 (fully activated). In the second scenario (Figure 12), the MR damper remained fully activated at the free end of the beam. At the same time the damage position L*_D_* was varied, and the intensity of the damage plasticity D was adjusted from 0 to 1, with 1 representing a fully broken beam.

In Figure 11, the maximum relative vibration energy (E*_R_*) occurs when the damage is positioned near the excitation force F(t) and the MR damper’s activation level is approximately 40%. While in Figure 12, the maximum E*_R_* is observed when the damage is located between the clamped end and the excitation force F(t), with the damage intensity remaining relatively low.

## 5. Conclusions

Smart materials, which adjust their properties in response to external stimuli like temperature, stress, and magnetic fields, hold considerable promise for various applications, including sensors, robotics, and adaptive structures. This research investigated such materials, specifically magnetic shape memory (MSM) alloys and magnetorheological (MR) fluids, to improve the dynamic control of beam-like structures.

It was found through finite element modelling simulations that integrating smart materials such as shape memory wires and magnetic devices can significantly reduce vibrations and enhance structural stability. The study reveals that the optimal placement of MSM actuators and MR dampers is crucial for maximizing vibration control. For an undamaged cantilever beam, MSM actuators are most effective when positioned between the excitation source and the free end, with an amplitude between 0.35 N and 1 N, while MR dampers achieve the highest vibration reduction with activation levels of 80% to 100% in the same region. When analysing a damaged beam, where a “plastic zone” simulates localized stiffness reduction, MSM actuators and MR dampers were shown to mitigate damage impacts effectively. For the MSM actuator, the greatest relative vibration energy reduction occurred when the actuator and damage zone were positioned between the excitation force and the free end. For the MR damper, effective vibration control was achieved with the damper fully activated and placed at the free end, regardless of the damage’s location or intensity. This comprehensive study demonstrates that the careful positioning and activation of MSM actuators and MR dampers are crucial for optimising structural performance and resilience under dynamic loading conditions.

Based on these numerical calculations, it is clear that active elements, such as SM wires, MSM actuators, and MR dampers, can effectively control, tune, or reduce forced vibrations in both undamaged and damaged beam-like structures. Success with these active elements depends on tailoring parameters for each specific case.

Future research should explore both theoretical and experimental applications of these active elements in more complex structures.

## Figures and Tables

**Table 1:** Composite material components’ properties.

	Matrix Epoxy	Glass Fibres
Elastic modulus [GPa]	3.43	66.5
Poisson ratio	0.35	0.23
Kirchhoff modulus G [GPa]	1.27	27.0
Density [kg/m^3^]	1250	2250

**Table 2:** SM wire, MSM actuator, and MR damper parameters (acc. to technical sheet from manufacturer [34,35,36]).

SM Wire			
Wire diameter [mm]		0.2–0.5	
Length [mm]		Can vary	
Temperature [°C]:			
	actuation	70–90	
	transformation	30–50	
Superelastic strain (%)		Up to 8	
Force output [N]		Up to 50–200 (depending on wire diameter)	
Recovery stress [MPa]		100–200	
Elastic modulus [GPa]:			
	austenite	70–90	
	martensite	20–40	
Density [kg/m^3^]		6450–6500	
**MSM Actuator**		**MR Damper**	
Piston dimensions [mm]	0.55 × 2.2 × 20	Piston diameter [mm]	10
Actuator dimensions [mm]	19 × 25 × 32	Damper diameter [mm]	42.1
Stroke [mm @ Hz]	0.6 @ 200	Stroke [mm]	55
Blocking force [N]	3	Tensile strength [N]	8896
Maximum frequency [Hz]	600	Damper forces [N]:	
		5 cm/sec@1A	>2447
		20 cm/sec@0A	>667
Max. peak current in coil [A]	1		

**Table 3:** Modes of a composite cantilever beam.

Mode					
**no. 1**	**no. 2**	**no. 3**	**no. 4**	**no. 5**	**no. 6**
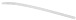	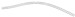	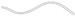	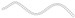	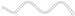	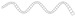

## Data Availability

The original contributions presented in the study are included in the article, further inquiries can be directed to the corresponding author.

## Abbreviations

The following abbreviations are used in this manuscript (alphabetical order):

$E_{R}$	Relative Vibration Energy
FEM	Finite Element Method
FSM	Ferromagnetic Shape Memory
M./m.	Material(s)/material(s)
MR	Magnetorheological
MSM	Magnetic Shape Memory
MSME	Magnetic Shape Memory Effect
PZT	Lead Zirconate Titanate (piezoelectric ceramic material)
SM	(thermal/classical) Shape Memory
SME	(thermal/classical) Shape Memory Effect
